# *DsDBF1*, a Type A-5 DREB Gene, Identified and Characterized in the Moss *Dicranum scoparium*

**DOI:** 10.3390/life13010090

**Published:** 2022-12-28

**Authors:** Alfred O. Onele, Anastasia B. Mazina, Ilya Y. Leksin, Farida V. Minibayeva

**Affiliations:** Kazan Institute of Biochemistry and Biophysics, FRC Kazan Scientific Center, P.O. Box 261, 420111 Kazan, Russia

**Keywords:** abiotic stress, dehydration-responsive element binding (DREB) transcription factors, gene expression, mosses, stress tolerance

## Abstract

Plant dehydration-responsive element binding (DREB) transcription factors (TFs) play important roles during stress tolerance by regulating the expression of numerous genes involved in stresses. DREB TFs have been extensively studied in a variety of angiosperms and bryophytes. To date, no information on the identification and characterization of DREB TFs in *Dicranum scoparium* has been reported. In this study, a new *DBF1* gene from *D. scoparium* was identified by cloning and sequencing. Analysis of the conserved domain and physicochemical properties revealed that DsDBF1 protein has a classic AP2 domain encoding a 238 amino acid polypeptide with a molecular mass of 26 kDa and a pI of 5.98. Subcellular prediction suggested that DsDBF1 is a nuclear and cytoplasmic protein. Phylogenetic analysis showed that DsDBF1 belongs to group A-5 DREBs. Expression analysis by reverse transcription quantitative real-time polymerase chain reaction (RT-qPCR) revealed that *DsDBF1* was significantly upregulated in response to abiotic stresses such as desiccation/rehydration, exposure to paraquat, CdCl_2_, high and freezing temperatures. Taken together, our data suggest that *DsDBF1* could be a promising gene candidate to improve stress tolerance in crop plants, and the characterization of TFs of a stress tolerant moss such as *D. scoparium* provides a better understanding of plant adaptation mechanisms.

## 1. Introduction

Abiotic stresses, such as drought, salinity, heavy metals, high and low temperatures, can disrupt cellular homeostasis, resulting in redox imbalances and the accumulation of reactive oxygen species (ROS), which can limit plant development and crop productivity [1,2]. To react and adapt to these environmental challenges, plants have developed complex mechanisms including physiological, biochemical, and molecular processes [3,4,5]. Significant progress has been made over the past two decades regarding the identification and characterization of stress-responsive genes and proteins that directly protect plants against stresses [5,6]. Numerous genes are regulated by transcription factors (TF) in response to various abiotic stimuli [7]. Transcription factors play important roles in controlling the expression of genes in various signaling pathways due to their DNA-binding specificity [8,9].

Dehydration-responsive element binding (DREB) TFs play critical roles in plant tolerance by regulating the expression of stress-inducible genes during abiotic stresses [10]. They have been extensively identified in a wide variety of higher plants (angiosperms), such as *Arabidopsis thaliana* [11], soybean (*Glycine max*) [12], rice (*Oryza sativa*) [13,14], maize (*Zea mays*) [15], barley (*Hordeum vulgare*) [16], and others. The dehydration-responsive element (DRE)-binding factor, also known as DBF1, belongs to the APETALA2/Ethylene-Responsive Factor (AP2/ERF) TF family, which has been demonstrated to be involved in various biological processes in plants, including metabolism, development, and stress response [12,17,18]. So far, AP2/ERF genes annotated in the mosses *Physcomitrium patens* and *Sphagnum fallax* are the largest TF families found in the plant TF databases (TFDB), although the AP2/ERF gene family has been rarely studied in the moss species [19,20,21]. Additionally, it has been shown that AP2/ERFs are regulated in response to numerous stresses, such as salinity and UV in *P. patens* [22] and the gene *PpDBF1* played a role in drought, salt, and cold tolerance in transgenic tobacco [23]. Furthermore, in the desiccation tolerant moss *Syntrichia caninervis*, AP2/ERFs were found to be the most abundant TFs [24].

*Dicranum scoparium* is a category “A” moss, one of the most desiccation tolerant moss species [25]. It is a widely distributed Holarctic moss that grows in various habitats and is one of the most polymorphic species in their genus [26]. Our preliminary analysis of class III peroxidase activity in three feather mosses such as *D. scoparium*, *Hylocomium splendens* and *Pleurozium schreberi* growing together in Aisha forest, Tatarstan, Russia revealed that they have high peroxidase activity and diverse peroxidase isoforms [27]. However, *D. scoparium* had the highest activity, approximately double that of *H. splendens* and *P. schreberi*, and this activity was stimulated by the desiccation/rehydration cycle. Therefore, *D. scoparium* was chosen for further investigation of desiccation tolerance mechanisms. Although to date the full genome of *D. scoparium* has not been sequenced and no reports of DREB families are available for this species, we have previously described in *D. scoparium* how temperature and desiccation/rehydration stresses change the expression of genes encoding Class I ascorbate peroxidase (*DsAPX*) and Class III peroxidases (*DsPODs*) [28]. We hypothesized that in Dicranum, abiotic stress will also influence the expression of *DBF1*. In this study, we report the isolation of a cDNA from *D. scoparium* that encodes a new DNA-binding TF, designated as DsDBF1. Furthermore, we analyzed the physico-chemical properties and subcellular localization of protein and gene expression patterns after desiccation/rehydration, high and low temperature, paraquat, DCMU, and CdCl_2_ stresses. Our study showed that *DsDBF1* was significantly upregulated after exposure of *D. scoparium* to abiotic stresses, especially desiccation/rehydration, freezing temperature, paraquat, and CdCl_2_, suggesting that this TF plays multiple roles in the tolerance of the moss to abiotic stresses.

## 2. Materials and Methods

### 2.1. Plant Material

*Dicranum scoparium* Hedw. was collected in the Aisha Forest in Tatarstan, Russia (55°53 21.3 N 48°38 14.3 E). Plant material was placed between sheets of paper and left to dry slowly in the open air for 2 days before being stored in the refrigerator at +4 °C in the dark until usage [28].

### 2.2. Identification and Retrieval of DsDBF1protein

Metatranscriptome data for the moss *D. scoparium* deposited to the Sequence Read Archive in the NCBI under accession numbers: PRJEB21674, ID: 393814 and PRJNA499105, ID: 499105 were extracted from the database [29,30]. The files were downloaded using the SRA Toolkit [31] and then converted to fastq format. FastQC software [32] was used to evaluate library quality control (QC). Adapter removal and trimming was done using Trimmomatic software version 0.39 [33]. After trimming, the reads were reassessed using the FastQC software [32]. Library assembly was performed using Trinity software [34]. All contaminants and foreign fungal and bacterial sequences were removed from the original data [29,30].

BLASTX [35] of representative sequences from mosses and other predicted taxa was used to determine the taxonomic classification of the identified transcripts (E-value < 1 × 10^−6^). To reduce transcript redundancy, moss transcripts containing the top hits were isolated to a separate file and filtered using the EvidentialGene package (https://sourceforge.net/projects/evidentialgene/, accessed on 10 October 2022) with the default parameters. The DBF1 amino acid sequence from *P. patens* was used as a query in a TBLASTN search [36] for a similar protein among the filtered transcripts from *D. scoparium*.

A DBF1 transcript was found after using TBLASTN and open reading frames (ORFs) were detected using the Augustus gene prediction and AssemblyPostProcessor tools in Galaxy version 1.0.3.0 (https://usegalaxy.org/, accessed on 10 October 2022). To confirm the domain-identifying members of the *DBF1* gene family, the predicted sequence was submitted against PFAM [37], NCBI Conserved Domains Database (CDD) [38], InterProScan [39], and HMMER [40].

### 2.3. Cloning and Sequencing of DBF1 Gene

Total RNA was extracted using the GeneJET Plant RNA Purification Mini Kit (Thermo Scientific, Vilnius, Lithuania). RNA concentration and purity were assessed using a NanoDrop^®^ ND-1000 spectrophotometer (Thermo Scientific, Waltham, MA, USA) and integrity was confirmed using 1% (*m/v*) agarose gel electrophoresis. First strand and double strand cDNA were synthesized using Evrogen Mint 2 synthesis kit according to manufacturer’s protocols.

To verify the *DBF1* from *D. scoparium* identified in silico, the *DBF1* sequence with the highest homology to *DBF1* from *P. patens* was cloned into the pAL2-T vector (Evrogen, Moscow, Russia) using primers: F TGGGTTCACACGATGCGGA; R ACGCTTTGAATCCACTGACGG and then sequenced.

### 2.4. Sequence Analysis

BLASTN software available online at (https://blast.ncbi.nlm.nih.gov/Blast.cgi, accessed on 12 October 2022) was used to perform a homology search to compare our sequenced *DsDBF1* with other genes in the database. Files in Fasta format were downloaded from the NCBI database after BLAST search and then subjected to multiple sequence alignments using Clustal Omega [41] and ClustalW [42] in MEGA X [43]. The Expasy ProtParam tool [44] was used to predict the physico-chemical properties of the DsDBF1 protein, including molecular weight, isoelectric point, instability index, and grand average of hydropathicity (GRAVY). Subcellular localization was predicted by MULocDeep [45].

The homologous sequences of DsDBF1 proteins obtained after BLASTX and other known DREB proteins from the NCBI database were aligned by ClustalW [42] in MEGA X [43]. A phylogenetic tree was constructed in MEGA X [43] using the neighbor joining method for 1500 bootstraps [46]. Evolutionary distances were calculated using the Poisson correction method [47] and all ambiguous positions were removed by pairwise deletion.

The MEME suite (http://meme-suite.org/index.html, accessed on 12 October 2022) was used to analyze DREB protein sequences to find conserved motifs with the following parameters: zero or one site per sequence, number of motifs (1–10), motif width (6–50) [48]. After MEME, the motif map was rebuilt using the TBtools software [49].

### 2.5. Stress Treatments

For stress treatment in this study, we followed the protocol developed in our early studies [28]. Before the experiment, 2 cm apical stem segments of dry mosses were pre-hydrated at +4 °C for 24 h on wet filter paper. For stress treatments, 0.2 g moss segments were incubated in 20 mL of 100 µM paraquat (1,1-dimethyl-4,4-bipyridylium dichloride), 100 µM DCMU (3-(3,4-dichlorophenyl)-1,1-dimethylurea), or 100 µM CdCl_2_ for 1 and 12 h. Hydrated apical stem segments were also thermally stressed by their exposure to −20 °C or +30 °C for 1 or 12 h in a dark temperature-controlled chamber (Thermostat LOIP, St. Petersburg, Russia). In all treatments, hydrated mosses kept at room temperature served as controls.

For desiccation stress, three biological replicates per treatment were used, each containing 0.17 g dry mass from 2 cm apical stem segments. Initially, air-dry mosses were fully hydrated by immersing them in a 20 mL volume of distilled water for 1 h while slowly shaking them on an orbital shaker. Then, the hydrated moss was gently blotted with filter paper and placed in the desiccator above silica gel. Here, moss samples were taken at time 0 (after 1 h of hydration), and after 2, 24, and 72 h of desiccation. After 72 h of desiccation, moss samples were rehydrated for 0.5 and 2 h. The change in relative water content (RWC) was monitored according to the protocol previously described in [28].

### 2.6. RNA Extraction, cDNA Synthesis and RT-qPCR

Samples exposed to stresses were immersed in liquid nitrogen, then, each sample was ground into a fine powder. For RT-qPCR, 0.1 g of material from each replicate was immediately frozen in liquid nitrogen and stored at −80 °C until use. Extraction of total RNA from *D. scoparium* thalli was performed using the RNeasy Plant Mini kit (Qiagen, Hilden, Germany) according to the manufacturer’s protocol. RNA concentration and purity were measured with NanoDrop^®^ ND-1000 spectrophotometer (Thermo Scientific, Waltham, MA, USA), and the integrity was further evaluated by gel electrophoresis in a 1% (*w/v*) agarose gel. First strand cDNA was synthesized using protocols from the Evrogen Mint 2 synthesis kit.

The vector NTI Suite 9 software was used to design RT-qPCR primers with the following parameters: amplicon length from 60 to 300 bp and a Tm range of 55 to 65 °C. RT-qPCR was performed on CFX Connect™ Real-Time System (Bio-Rad Laboratories, Singapore) with qPCRmix-HS SYBR (Evrogen). The templates were amplified three times at 95 °C for 3 min followed by 40 cycles of amplification (94 °C for 10 s and 55/60 °C for 40 s). Melting curve analysis after RT-qPCR and gel electrophoresis examination of the amplified products were used to assess the specificity of the primers. The gene-specific primers used for RT-qPCR are listed in Table S1. Ribosomal RNA (*18S*), glyceraldehyde-3-phosphate dehydrogenase (*GAPDH2*) and α-tubulin (*α-TUB1* and *α-TUB2*) were used as internal controls for RT-qPCR normalization [28].

### 2.7. Statistical Analysis

Three biological and six analytical replicates were used to run all reactions. Gene expression differences were assessed using normalized expression (Cq) in the Bio-Rad CFX Maestro^TM^/Software version 2.3 and were found to be significant for *p* ≤ 0.05 (*), *p* ≤ 0.01 (**), *p* ≤ 0.001 (***) after ANOVA and Shapiro–Wilk Normality tests. The standard errors of the mean are shown as vertical bars (n = 6).

## 3. Results

### 3.1. Characterization and Phylogenetic Analysis of DsDBF1

**Figure 1:**
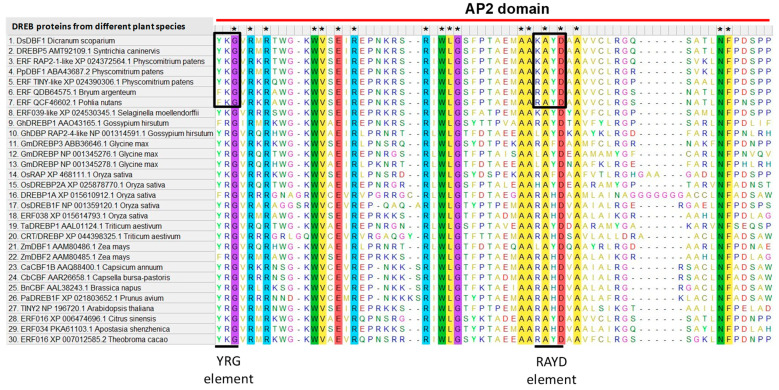
Sequence alignments of DsDBF1 and other known ERF/DREB proteins showing the classical AP2 domains from mosses and vascular plants such as *Syntrichia caninervis* (DREBP5_AMT92109.1), *Physcomitrium patens* (ERF RAP2-1-like_XP 024372564.1; PpDBF1 ABA43687.2; ERF_TINY-like_XP 024390306.1), *Gossypium hirsutum* (GhDREBP1_AAO43165.1; GhDBP_RAP2-4-like_NP 001314591.1), *Glycine max* (GmDREBP3_ABB36646.1; GmDREBP_NP 001345276.1; GmDREBP_NP 001345278.1), *Oryza sativa* (OsRAP_XP 468111.1; OsDREBP2A_XP 025878770.1; DREBP1A_XP 015610912.1; OsDREB1F_NP 001359120.1; ERF038_XP 015614793.1), *Triticum aestivum* (TaDREBP1_AAL01124.1; CRT/DREBP_XP 044398325.1), *Zea mays* (ZmDBF1_AAM80486.1; ZmDBF2_AAM80485.1), *Capsicum annuum* (CaCBF1B_AAQ88400.1), *Capsella bursa-pastoris* (CbCBF_AAR26658.1), *Brassica napus* (BnCBF_AAL38243.1), *Prunus avium* (PaDREB1F_XP 021803652.1), *Arabidopsis thaliana* (TINY2_NP 196720.1), *Bryum argenteum* (ERF_QDB64575.1), *Pohlia nutans* (ERF_QCF46602.1), *Selaginella moellendorffii* (ERF039-like_XP 024530345.1), *Citrus sinensis* (ERF016_XP 006474696.1), *Apostasia shenzhenica* (ERF034_PKA61103.1), and *Theobroma cacao* (ERF016_XP 007012585.2). Amino acid sequences are highlighted with different colors. Sequences marked by (*) show conserved amino acid residues. Two conserved elements (YRG and RAYD) are marked by black horizontal lines. The differences in the moss conserved elements are shown in black frames. The red line shows the classical AP2 domain.

A *DBF1* gene was identified based on the metatranscriptome data for the moss *D. scoparium* downloaded from the Sequence Read Archive in the NCBI. To verify the *DBF1* gene identified from *D. scoparium*, specific primers were designed and the PCR product (717 bp) was cloned into the pAL2-T vector (Evrogen, Moscow, Russia) and then sequenced. Blasting the *DBF1* sequence from *D. scoparium* after cloning and sequencing revealed high homology with *ERF/DREBs* of other mosses and vascular plants in the NCBI database. Further analyses of the protein sequence using PFAM [37], NCBI CDD [38], InterProScan [39] and HMMER [40] databases revealed that this protein had a classic AP2 domain structure (Figure 1) and was named DsDBF1. In addition, coding domain sequence (CDS) length (bp), subcellular localization, and physico-chemical properties such as protein length (aa), molecular weight (MW, kDa), isoelectric point (pI), instability index, and GRAVY were predicted (Table 1). The results showed that the cloned *DsDBF1* encoded a 238 amino acid polypeptide (Figure S1) with a predicted molecular weight of 26 kDa and isoelectric point of 5.98. Calculation of the instability index classified the protein as unstable with a value greater than 40. A negative value of GRAVY indicated that DsDBF1 was hydrophilic and subcellular prediction showed that the protein was localized within the nucleus and cytoplasm (Table 1).

**Figure 2:**
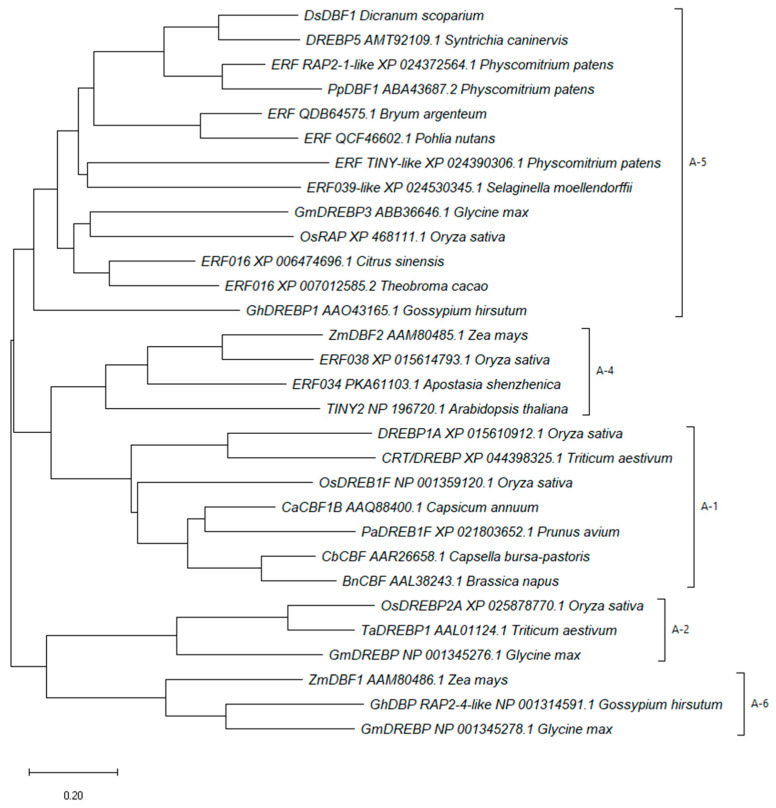
Phylogenetic analyses of DsDBF1 and other ERF/DREB proteins from mosses and vascular plant constructed using the neighbor-joining method with 1500 bootstrap test showing the relationship between the amino acid sequences. Evolutionary distances were calculated using the Poisson correction method and all ambiguous positions were removed by pairwise deletion. Amino acid sequences used for phylogenetic tree construction were retrieved, in part, from GenBank and after blast analysis from GenBank: *Syntrichia caninervis* (DREBP5_AMT92109.1), *Physcomitrium patens* (ERF RAP2-1-like_XP 024372564.1; PpDBF1 ABA43687.2; ERF_TINY-like_XP 024390306.1), *Gossypium hirsutum* (GhDREBP1_AAO43165.1; GhDBP_RAP2-4-like_NP 001314591.1), *Glycine max* (GmDREBP3_ABB36646.1; GmDREBP_NP 001345276.1; GmDREBP_NP 001345278.1), *Oryza sativa* (OsRAP_XP 468111.1; OsDREBP2A_XP 025878770.1; DREBP1A_XP 015610912.1; OsDREB1F_NP 001359120.1; ERF038_XP 015614793.1), *Triticum aestivum* (TaDREBP1_AAL01124.1; CRT/DREBP_XP 044398325.1), *Zea mays* (ZmDBF1_AAM80486.1; ZmDBF2_AAM80485.1), *Capsicum annuum* (CaCBF1B_AAQ88400.1), *Capsella bursa-pastoris* (CbCBF_AAR26658.1), *Brassica napus* (BnCBF_AAL38243.1), *Prunus avium* (PaDREB1F_XP 021803652.1), *Arabidopsis thaliana* (TINY2_NP 196720.1), *Bryum argenteum* (ERF_QDB64575.1), *Pohlia nutans* (ERF_QCF46602.1), *Selaginella moellendorffii* (ERF039-like_XP 024530345.1), *Citrus sinensis* (ERF016_XP 006474696.1), *Apostasia shenzhenica* (ERF034_PKA61103.1), and *Theobroma cacao* (ERF016_XP 007012585.2).

Sequence alignment analysis indicated that DsDBF1 shared high homology and a conserved AP2/ERF domain with other DREBs (Figure 1), but with low similarity in their overall amino acid sequences (Figure S2). Additionally, two conserved elements (YRG and RAYD) were found in the AP2/ERF domain after sequence analysis, although arginine (R) is replaced by lysine (K) in both the first YRG and second RAYD elements in DsDBF1 and some DREBs of other mosses (Figure 1). The homologous protein sequences obtained after BLASTP search of the DsDBF1 sequence and other known ERF/DREB proteins from GenBank were used to construct a phylogenetic tree demonstrating the evolutionary relationship between DsDBF1 and other similar sequences from mosses and vascular plants. The evolutionary tree showed that DsDBF1 belongs to the A-5 group of the DREB subfamily as it shared a common ancestry and homology with other known A-5 DREBs from mosses such as *S. caninervis*, *P. patens, Bryum argenteum*, *Pohlia nuntans,* and vascular plants such as *Selaginella moellendorffii*, *G. max*, *O. sativa*, *Citrus sinensis*, *Theobroma cacao,* and *Gossypium hirsutum* (Figure 2). Furthermore, it was found that group A-5 was divided into seven subgroups, with *S. moellendorffii* positioning between the protein subgroups of mosses and the vascular plants. As shown in Figure 2, all other known DREBs from vascular plants were clustered into different DREB subfamilies such as A-1, A-2, A-4, and A-6.

**Figure 3:**
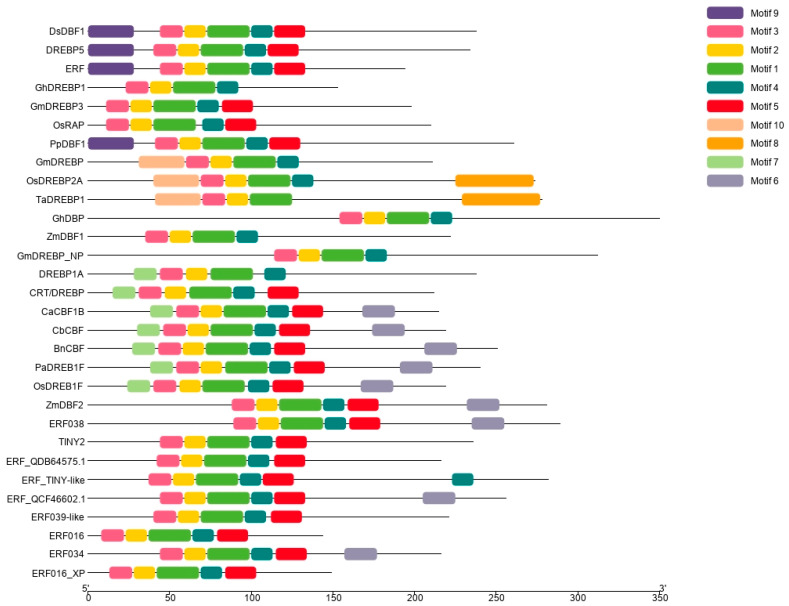
Motif analysis of DsDBF1 and other known classic ERF/DREB proteins from mosses and vascular plants: *Syntrichia caninervis* (DREBP5_AMT92109.1), *Physcomitrium patens* (ERF RAP2-1-like_XP 024372564.1; PpDBF1 ABA43687.2; ERF_TINY-like_XP 024390306.1), *Gossypium hirsutum* (GhDREBP1_AAO43165.1; GhDBP_RAP2-4-like_NP 001314591.1), *Glycine max* (GmDREBP3_ABB36646.1; GmDREBP_NP 001345276.1; GmDREBP_NP 001345278.1), *Oryza sativa* (OsRAP_XP 468111.1; OsDREBP2A_XP 025878770.1; DREBP1A_XP 015610912.1; OsDREB1F_NP 001359120.1; ERF038_XP 015614793.1), *Triticum aestivum* (TaDREBP1_AAL01124.1; CRT/DREBP_XP 044398325.1), *Zea mays* (ZmDBF1_AAM80486.1; ZmDBF2_AAM80485.1), *Capsicum annuum* (CaCBF1B_AAQ88400.1), *Capsella bursa-pastoris* (CbCBF_AAR26658.1), *Brassica napus* (BnCBF_AAL38243.1), *Prunus avium* (PaDREB1F_XP 021803652.1), *Arabidopsis thaliana* (TINY2_NP 196720.1), *Bryum argenteum* (ERF_QDB64575.1), *Pohlia nutans* (ERF_QCF46602.1), *Selaginella moellendorffii* (ERF039-like_XP 024530345.1), *Citrus sinensis* (ERF016_XP 006474696.1), *Apostasia shenzhenica* (ERF034_PKA61103.1), and *Theobroma cacao* (ERF016_XP 007012585.2). Distribution of 10 putative conserved motifs in DREB proteins is shown. Conserved motifs are represented by different colored boxes numbered 1–10.

Additionally, the results of MEME analyses showed that DsDBF1 contained a total of six motifs, among them, motifs 1–3 represented the basic conserved motifs that made up the AP2 domain (Figure 3). Motif 4 was absent only in the DREB protein of *Triticum aestivum* (TaDREBP1_AAL01124.1), while motif 5 was absent in the DREB proteins of *Gossypium hirsutum* (GhDREBP1 AAO43165.1 and GhDBP RAP2-4-like_NP 001314591.1), *G. max* (GmDREBP3 ABB36646.1 and GmDREBP_NP 001345278.1), *O. sativa* (OsRAP_XP 468111.1 and DREBP1A_XP 015610912.1), *Z. mays* (ZmDBF1_AAM80486.1) including *T. aestivum* (TaDREBP1_AAL01124.1). However, an additional motif 9 was detected in DsDBF1, which was only conserved in *S. caninervis* (DREBP5_AMT92109.1) and two in *P. patens* (ERF RAP2-1-like_XP 024372564.1 and PpDBF1_ABA43687.2) (Figure 3).

### 3.2. Expression Patterns of DsDBF1 in Response to Abiotic Stress Treatments

**Figure 4:**
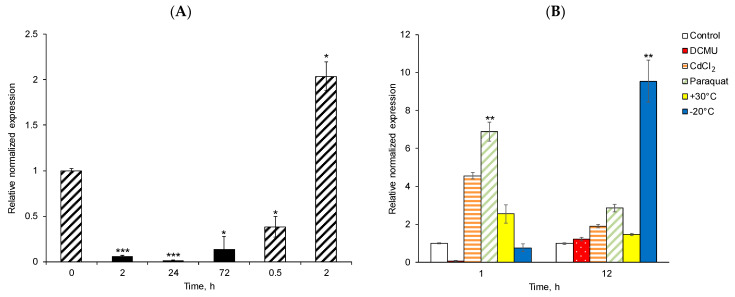
Expression patterns of *DsDBF1* under abiotic stress treatments analyzed using RT-qPCR. (**A**) Relative expression of *DsBF1* during desiccation over silica gel and rehydration. Shaded bars represent the hydrated and rehydrated moss, and solid bars represent the desiccated moss. (**B**) Relative expression of *DsDBF1* exposed to DCMU, CdCl_2_, paraquat and high/low temperature for 1 and 12 h. Open bars correspond to control samples of mosses kept at room temperature. Red bars with white dots represent mosses treated with 100 µM DCMU, bars with orange horizontal stripes show moss treated with 100 µM CdCl_2_, bars with green stripes correspond to samples subjected to 100 µM paraquat, yellow and blue bars correspond to mosses exposed to +30 °C and −20 °C, respectively. *p* ≤ 0.05 (*), *p* ≤ 0.01 (**), *p* ≤ 0.001 (***). The vertical bars indicate the standard errors of the mean (n = 6).

The expression pattern of *DsDBF1* was studied after application of abiotic stresses such as desiccation/rehydration, exposure to DCMU, CdCl_2_, paraquat, high and freezing temperatures to moss apical segments. Desiccation of the hydrated moss for 2, 24, and 72 h over silica gel resulted in almost up to 94% loss of RWC in the moss samples, accompanied by downregulation of *DsDBF1*, with the lowest expression observed after 24 h of dehydration (Figure 4A). Rehydration of the mosses after 72 h of desiccation showed a gradual increase in *DsDBF1* expression after 0.5 h and further 2 h with the expression of *DsDBF1* 2-fold higher compared to that in the hydrated mosses before desiccation (Figure 4A). Treatment of moss segments with an inhibitor of photosynthesis DCMU downregulated *DsDBF1* expression after 1 and 12 h (Figure 4B). Subjecting the mosses to heavy metal CdCl_2_ and prooxidant paraquat significantly increased the expression of *DsDBF1* after 1 h (Figure 4B); however, further treatment for 12 h downregulated *DsDBF1* expression. No significant changes in *DsDBF1* expression were observed after exposing moss to +30 °C for 1 and 12 h. Exposure of mosses to a freezing temperature of −20 °C reduced the level of *DsDBF1* expression after 1 h (Figure 4B); however, further exposure for 12 h at −20 °C upregulated gene expression almost 10-fold compared to a 1 h treatment (Figure 4B).

## 4. Discussion

Members of the AP2/ERF family of TFs are among the most important key regulators of genes responsible for stress tolerance and developmental transitions of plants. These TFs regulate transcriptional networks to activate or repress gene expression in response to biotic and abiotic factors through the modulation of several signaling pathways [7,8,17,50]. In the last few years, many DREBs have been identified and characterized in several angiosperms, including *A. thaliana* [11], rice (*O. sativa*) [13,51], soybean (*G. max*) [52], maize (*Z. mays*) [15,53], cotton (*G. hirsutum*) [54,55], barley (*H. vulgare*) [16,56,57], wheat (*T. aestivum*) [58], *Populus euphratica* [59], *Caragana korshinskii* [60], and others. Surprisingly, the AP2/ERF gene family has been rarely studied in stress-tolerant moss species [19,20,21]. Several recent studies have shown that AP2/ERF TFs play an important role in the developmental processes and stress responses in some moss species, such *P. patens* [22,23,61], *S. caninervis* [24,62,63,64,65], *B. argenteum* [21], and *P. nutans* [66]. *Dicranum scoparium* is a desiccation-tolerant moss [25] whose genome has not been fully sequenced, and no TF families of this species have been reported to date. In this present study, we first identified in silico a cDNA of the *DBF1* gene in the moss *D. scoparium*. Then, the identified gene was verified by cloning and sequencing. In addition, we performed molecular characterization of the protein, including analysis of the conserved domain, physico-chemical properties, subcellular localization, phylogenetic relationship, and motif analyses of identified DsDBF1 and DREBs of other plants, and finally, we examined the expression patterns of this gene in response to abiotic stresses. Our results demonstrate that the expression of *DsDBF1* is strongly induced by rehydration after desiccation, and treatments with CdCl_2_, paraquat, and freezing temperature, providing insights into the roles of *DBF1* in response of *D. scoparium* to abiotic stresses.

Analyses of the physico-chemical properties and the subcellular localization showed that *DsDBF1* encodes a 238-amino acid polypeptide with a molecular weight of 25 kDa and a pI of 5.98 and the protein is localized within the nucleus and cytoplasm (Table 1). While the majority of TFs are nuclear localized, some are not when initially synthesized [67]. Some of these TFs are kept inactive in the cytoplasm when synthesized or expressed as membrane proteins, but when stimulated, they are activated by proteolytic cleavage, releasing the active form, which enters the nucleus and activates target genes [67,68].

Furthermore, the BLASTP search of the NCBI database revealed that DsDBF1 shares high sequence similarities with some DREBs from angiosperms and mosses. In addition, some uncharacterized proteins from mosses such as *Ceratodon purpureus* and *S. fallax* also show very high similarities to DsDBF1. The amino acid composition of the AP2 domain of DsDBF1 revealed that it contains 65 amino acid residues (Figure 1), which approximately corresponds to the conserved 60 amino acids of the AP2/ERF domain found in all DREBs [11]. Amino acid alignments of DREB proteins from different plants show high sequence similarity in the middle of AP2/ERF domain of these proteins (Figure 1), which is a significant feature of plant DREBs [64,69]. However, in general, outside the domain box, low similarity is observed in their overall amino acid sequences (Figure S2).

Analysis of the AP2/ERF domain after multiple sequence alignments revealed the presence of two conserved YRG and RYAD elements (Figure 1), although only glycine (G) is conserved in the YRG element among all the DREBs, while alanine (A) and aspartic acid (D) are conserved in the RYAD elements (Figure 1). Furthermore, in the first YRG element, tyrosine (Y) and arginine (R) are replaced by phenylalanine (F) and lysine (K), respectively, whereas in the second RYAD element, R is substituted by K, leucine (L), and histidine (H), and the Y is substituted by F and H. The AP2/ERF domain is a type of DNA-binding module that contains two known conserved elements (YRG and RAYD), and these two elements can bind with the promoter sequence or some other interacting proteins [69,70]. Studies have shown that YRG is involved in DNA binding activity and is the basic hydrophilic N-terminal side of the AP2/ERF domain. The N-terminal region is approximately 19 to 22 amino acids in length [50,69,70]. In addition, the second element, RAYD, is located in the acidic C-terminal region of the AP2/EREBP domain with a length of 42 to 43 amino acids. It is suggested that the RAYD element plays a crucial role in mediating protein–protein interactions [50,70]. However, in this study, the substitution of amino acids observed at various positions within the conserved elements in the AP2/ERF domain after multiple sequence alignments (Figure 1) of DREB proteins, may imply their functional divergence within DREB subfamilies.

To understand the evolutionary relationship between DsDBF1 and other well-known DREBs from other plants, a neighbor-joining tree was constructed using the deduced amino acid residues of these DREB proteins (Figure 2). In this analysis, DsDBF1 was found to belong to the A-5 group of the DREB subfamily as it shares a common ancestor with other known A-5 DREBs from mosses such as *S. caninervis*, *P. patens*, *B. argenteum*, *P. nuntans*, a lycophyte, for example, *S. moellendorffii*, and the angiosperms, such as *G. max*, *O. sativa*, *C. sinensis*, *T. cacao,* and *G. hirsutum* (Figure 2). In the A-5 subgroup, *S. moellendorffii* branches from the moss subgroup, positioning itself between the mosses and the angiosperms. This supports the report of early divergence of vascular plants from the ancient non-vascular plants [71]. It has been proposed that *PpDBF1*, an A-5 type DREB from *P. patens*, is an ancestor of DREB proteins and plays a general role in various stresses in non-vascular moss, which has diverged into different subclasses with different functions in the higher plants [23]. Consequently, the grouping of DsDBF1 and some other A-5 DREB proteins from mosses, lycophytes, and angiosperms in one clade suggests that they were established in the early stages of land plant evolution. Additionally, it was found that all other known DREBs from vascular plants diverged into different DREB subfamilies such as A-1, A-2, A-4, and A-6 (Figure 2). The DREB gene subfamily may have evolved and assumed new roles as a result of the divergence of the AP2 genes. The functional diversity and divergence of DREB genes during the adaptive evolution of stress signaling pathways in plants is most likely the result of subsequent duplication and transposition events [23].

Moreover, an investigation of the conserved motifs in DsDBF1 and other selected DREB proteins was carried out using MEME software. From the results, DsDBF1 contains a total of six motifs. Motifs 1–3 represent the conserved motifs of the AP2 domain (Figure 3). Furthermore, an additional motif 9 was detected in DsDBF1. This motif is present in *S. caninervis* (DREBP5_AMT92109.1) and *P. patens* (ERF RAP2-1-like_XP 024372564.1 and PpDBF1_ABA43687.2) (Figure 3), suggesting their common origin. Genome-wide sequence analysis of AP2/ERF family TFs in numerous plants revealed conserved regions and motifs on both sides of the AP2/ERF domain with important roles in transcriptional activity, protein–protein interactions, and nuclear localization. These conserved motifs can serve as an evidence for further classification of subgroups [50,72].

Plant DREB TFs play critical roles in the response to dehydration, salinity, and cold stresses [73,74]. To further understand the role of *DsDBF1* in response to stresses, we examined the expression profile of *DBF1* gene by RT-qPCR in the *D. scoparium* subjected to desiccation/rehydration, exposure to DCMU, CdCl_2_, paraquat, heat and freezing temperature. Our results indicate that *DsDBF1* gene is upregulated by most of these stresses, suggesting that this gene is involved in *D. scoparium* response to abiotic stresses (Figure 4A,B). Surprisingly, *DsDBF1* gene is downregulated following exposure of the moss to DCMU (Figure 4B). It has been reported that genes assigned to different groups within the same gene family show diverse stress response patterns and stress tolerance [64,75]. To date, most reports on DREB and Cold binding factors (CBFs) have mainly focused on DREBA1 and DREBA2, the largest among the subgroups [69,76]. The A-1 type DREBs (DREB1) are induced by cold and improve plant stress tolerance to low temperatures [73,77], whereas A-2 type DREBs (DREB2) play a major role in response to dehydration and heat stress, and improve drought and salt tolerance in plants [78]. A-5 DREBs have rarely been studied, and the functional and stress response mechanisms are still unclear [63].

Interestingly, desiccation of the hydrated mosses for 2, 24, and 72 h decreased *DsDBF1* expression (Figure 4A). However, rehydrating moss thalli after 72 h of desiccation progressively increased *DsDBF1* expression to 2-fold higher compared to the hydrated mosses before desiccation (Figure 4A). *PpDBF1*, a homolog of *DsDBF1,* was weakly induced by dehydration stress but strongly induced by ABA [23]. Out of ten A-5 type DREBs from *S. caninervis*, *ScDREB5* was downregulated under rapid desiccation stress over silica gel, while *ScDREB3*, *ScDREB9,* and *ScDREB10* were poorly induced by desiccation [63]. However, these four DREBs were significantly induced by cold stress, while *ScDREB3* and *ScDREB5* were upregulated during heat stress [63]. The rapid desiccation used in our experiment, in which moss thalli were dried over silica gel and reached an RWC of 6% [28] after 24 h, never occurs in boreal forests, where drying rates of mosses are much slower [27,79]. Other A-5-type DREBs such as *GhDBP1* and *GmDREB3* have been shown to improve plant stress tolerance [23,55,77]. Moreover, photosynthesis inhibitor DCMU decreased *DsDBF1* expression (Figure 4B). Meanwhile, CdCl_2_ and paraquat significantly altered the expression of *DsDBF1*, as short-term exposure increased *DsDBF1* expression after 1 h (Figure 4B). Long-term treatment of moss samples with paraquat and CdCl_2_ resulted in downregulation of *DsDBF1* expression after 12 h. Furthermore, exposure of moss to +30 °C had little effect on *DsDBF1* expression, although a freezing temperature of −20 °C for 12 h upregulated gene expression almost 10-fold compared to a 1 h cold treatment (Figure 4B). It has been reported that *PpDBF1*, *GmDREB2*, *StDREB2, ScDREB1*, *ScDREB2*, *ScDREB4*, *ScDREB6*, *ScDREB7,* and *ScDREB8* responded to drought, salt, and cold treatment among members of the A-5 subgroups [23,63,80,81]. Taken together, the upregulation of *DsDBF1* during rehydration after desiccation, exposure to CdCl_2_, paraquat, and freezing-temperature stress suggests that *DsDBF1,* like other A-5 DREBs, plays important roles in *D. scoparium* stress tolerance.

## 5. Conclusions

An A-5 type gene, *DsDBF1*, encoding DRE-binding transcription factor TF was identified and cloned in the moss *D. scoparium*. *DsDBF1* protein was predicted to be localized within the nucleus and cytoplasm. Furthermore, RT-qPCR analysis showed that *DsDBF1* expression was significantly induced in response to abiotic stresses such as desiccation/rehydration, exposure to paraquat, CdCl_2_, high and freezing temperatures. *D. scoparium* is a desiccation tolerant moss species. Based on our results, we believe that *DsDBF1* could be a promising gene candidate to improve stress tolerance in various crop plants, and characterization of transcription factors of a stress-tolerant moss such as *D. scoparium* provides a better understanding of plant response and adaptation mechanisms.

## Figures and Tables

**Table 1:** Physico-chemical properties and subcellular localization of DsDBF1.

Parameters	DsDBF1
CDS length, bp	717
Number of amino acids	238
Molecular weight (kDa)	26
Theoretical pI	5.98
Instability index	64.99
Grand average of hydropathicity (GRAVY)	−0.224
Subcellular localization prediction	Nucleus/cytoplasm

## Data Availability

Not applicable.

## Supplementary Materials

The following supporting information can be downloaded at: https://www.mdpi.com/article/10.3390/life13010090/s1, Figure S1: *DsDBF1* coding domain sequence and protein sequence; Figure S2: Sequence alignments of DsDBF1 and other known ERF/DREB proteins from mosses and vascular plants such as *Syntrichia caninervis* (DREBP5_AMT92109.1), *Physcomitrium patens* (ERF RAP2-1-like_XP 024372564.1; PpDBF1 ABA43687.2; ERF_TINY-like_XP 024390306.1), *Gossypium hirsutum* (GhDREBP1_AAO43165.1; GhDBP_RAP2-4-like_NP 001314591.1), *Glycine max* (GmDREBP3_ABB36646.1; GmDREBP_NP 001345276.1; GmDREBP_NP 001345278.1), *Oryza sativa* (OsRAP_XP 468111.1; OsDREBP2A_XP 025878770.1; DREBP1A_XP 015610912.1; OsDREB1F_NP 001359120.1; ERF038_XP 015614793.1), *Triticum aestivum* (TaDREBP1_AAL01124.1; CRT/DREBP_XP 044398325.1), *Zea mays* (ZmDBF1_AAM80486.1; ZmDBF2_AAM80485.1), *Capsicum annuum* (CaCBF1B_AAQ88400.1), *Capsella bursa-pastoris* (CbCBF_AAR26658.1), *Brassica napus* (BnCBF_AAL38243.1), *Prunus avium* (PaDREB1F_XP 021803652.1), *Arabidopsis thaliana* (TINY2_NP 196720.1), *Bryum argenteum* (ERF_QDB64575.1), *Pohlia nutans* (ERF_QCF46602.1), *Selaginella moellendorffii* (ERF039-like_XP 024530345.1), *Citrus sinensis* (ERF016_XP 006474696.1), *Apostasia shenzhenica* (ERF034_PKA61103.1) and *Theobroma cacao* (ERF016_XP 007012585.2). Multiple alignment was performed using Clustal Omega. Amino acid sequences are highlighted with different colors. Sequences marked by (*) show conserved amino acid residues, Table S1: Primers of RT-qPCR.
