# Supplementary material for: DsDBF1, a Type A-5 DREB Gene, Identified and Characterized in the Moss Dicranum scoparium
Source: Life (Basel). 2022 Dec 28;13(1):90. doi: 10.3390/life13010090 (PMC9862540; doi:10.3390/life13010090)
Supplement: Supplementary file 1 [file life-13-00090-s001.zip › Figure S1.pdf]

DsDBF1 Coding domain sequence (CDS)

atg gta gaa aag acg gct ccg tcg tca att agt gcc aag cgg ggc gga
aag ctc ttg gcg cct ctg aaa acg cac tcc ggg gcc ata gcc aag aaa
tcc tct gca ccg gag aaa ctg tcc att tcg caa tcg ccc gcg tgt ccg
aag ctg tac aag ggc gtg cgc atg cgc acg tgg ggg aaa tgg gtg tct
gag att cga gag ccc aac aag agg tcg cgc att tgg ctg ggc tcc ttc
ccc acg gcc gaa atg gca gcc aaa gcc tac gac gcg gcc gtg gtg tgc
ctc cga ggc cag tcc gcg acg ctc aat ttc ccc gac tca ccc ccg cag
tgc atc tcc ccc tcc cga gct ccc aag gac gtg caa gca gcc gcg gcc
gcc gct gcg gct gcg tgc gcc tcc gcc tcc cct ctc gca gag ccc acc
aac acc ccc aca ttc gag agc acc acc gtc gaa tcg ttg cac tcg ccc
ctg cat tgc gaa gca tcg tcc gcg ctc gcc atg gcc gag ccg cag tct
agc ttc gag gtg gag gag tgg atc caa gcg gag ttc gga gac ttg gag
ccc ctg atc gac aac gcc ttc cgc ttc cct gag ctc ccg ccg tgc gta
ttc gac tcc cag ttt caa atc ttc caa ccc agc gcc gga ccc ctc gag
gcg gac aac aga gcg ctc tac gac agc ctt tgg tgc ttt tcg taa

DsDBF1 protein sequence

MVEKTAPSSISAKRGGKLLAPLKTHSGAIAKKSSAPEKLSISQSPACPKLYKGVRMRTWGKWVS
EIREPNKRSRIWLGSFPTAEMAAKAYDAAVVCLRGQSATLNFPDSPPQCISPSRAPKDVQAAAA
AAAAACASASPLAEPTNTPTFESTTVESLHSPLHCEASSALAMAEPQSSFEVEEWIQAEFGDLE
PLIDNAFRFPELPPCVFDSQFQIFQPSAGPLEADNRALYDSLWCFS
