# Supplementary material for: DsDBF1, a Type A-5 DREB Gene, Identified and Characterized in the Moss Dicranum scoparium
Source: Life (Basel). 2022 Dec 28;13(1):90. doi: 10.3390/life13010090 (PMC9862540; doi:10.3390/life13010090)
Supplement: Supplementary file 1 [file life-13-00090-s001.zip › Figure S2.pdf]

```
GmDREBP            ------------------------------------------------------------   0
OsDREBP2A          ---------------------------------ME-------------------------   2
TaDREBP1           --------------------------------MET-------------------------   3
DREBP1A            ------------------------------------------------------------   0
CRT/DREBP          ------------------------------------------------------------   0
OsDREB1F           --------------------------------MDT-------------------------   3
CbCBF              ---------------------MNSSFSAFSEMF---------------------------   12
BnCBF              ----------------------MNSVSTFSELL---------------------------   11
CaCBF1B            ---------------------MNIFRSYYSDPLTE-------------------------   14
PaDREB1F           ---------------------MDMFFSQLSDSVDQ-------------------------   14
GhDBP              MAAAMDF---ISIGVDQSDLYGGELMEALEPFMKSVSSSSPSPSPSPSPSSLPSTSYLSF   57
GmDREBP_NP         MGTAIDMYNSSNIVADFLDPYSEELMKALKPFMKSDYFSASSSSSL--------------   46
ZmDBF1             ------------------------------------------------------------   0
TINY2              --------------------------------MAEEYYSL--------------------   8
ERF034             --------------------------------MVEPLS------PT--------------   8
ZmDBF2             --------------------------------MAQELHETSSCSAT--------------   14
ERF038             ------------------------------MAMAKELQETSSSSS---------------   15
GhDREBP1           ------------------------------------------------------------   0
ERF_QDB64575.1     --------------------------------MVD---TPRSS-----------------   8
ERF_QCF46602.1     --------------------------------MVDKQRLHTSS-----------------   11
ERF                --------------------------------MVEK--IAVSS-----------------   9
PpDBF1             --------------------------------MVEK--VVVSS-----------------   9
DsDBF1             --------------------------------MVEK--TAPSS-----------------   9
DREBP5             --------------------------------MVEK--AVSST-----------------   9
ERF039-like        ---------------------MGESIGVVAEQQPTT------------------------   15
OsRAP              ------------------------------------------------------------   0
ERF_TINY-like      --------------------------------MVQK------------------------   4
GmDREBP3           ------------------------------------------------------------   0
ERF016             ------------------------------------------------------------   0
ERF016_XP          ------------------------------------------------------------   0

GmDREBP            -----------------------------MHMLVK-NHNKGDGSKSLADTLAKW------   24
OsDREBP2A          -----RGEGRRGD------------CSVQVRKKR-TRRKSDGPDSIAETIKWW------   37
TaDREBP1           -----GGSKREGD------------CPGQERKKK-VRRRSTGPDSVAETIKKW------   38
DREBP1A            ------------------------------------------------------------   0
CRT/DREBP          ------------------------------------------------------------   0
OsDREB1F           ------------------------------------------------------------   3
CbCBF              ------------------------------------------------------------   12
BnCBF              ------------------------------------------------------------   11
CaCBF1B            ------------------------------------------------------------   14
PaDREB1F           ------------------------------------------------------------   14
GhDBP              SSSETQPNFYPDSCCYPYPTPMDSVSCPQQPQTGSTIGLNSLTQAQIHQIQLQFHLHNNQ   117
GmDREBP_NP         ---ESQ------PCSF------SSNSLPTSYPSSNQIKLNQLTPDQIVQIQAQIHIQQQQ   91
ZmDBF1             ----------------------------------------------MQFIQAQLHLQRNP   14
TINY2              -------------------------------------RS---------------------   10
ERF034             -----SSSACSSSCLPTS------------------------------------------   21
ZmDBF2             -----TTSSCTTSCCSSTVT--DSSSSPPSPAAANAAPA---------------------   46
ERF038             ------SSAASTSSCSSAVT--DAWSSPARPNAVAGGK----------------------   45
GhDREBP1           --------------------------------------M---------------------   1
ERF_QDB64575.1     ------------------------------------PVS---------------------   11
ERF_QCF46602.1     ------------------------------------PSR---------------------   14
ERF                ------------------------------------LHA---------------------   12
PpDBF1             ------------------------------------LRR---------------------   12
DsDBF1             ------------------------------------ISA---------------------   12
DREBP5             ------------------------------------LRG---------------------   12
ERF039-like        ------------------------------------IWD---------------------   18
OsRAP              ------------------------------------------------------------   0
ERF_TINY-like      ------------------------------------DWS---------------------   7
GmDREBP3           ------------------------------------------------------------   0
ERF016             ------------------------------------------------------------   0
ERF016_XP          ------------------------------------------------------------   0
```

```
GmDREBP              -----------------KEYNAWLESNNE---------AEKPVRKVPAKGSKKGCMKGKG     58
OsDREBP2A            -----------------KEQNQKLQEE-------------NSSRKAPAKGSKKGCMAGKG     67
TaDREBP1             -----------------KEENQKLQQE-------------NGSRKAPAKGSKKGCMAGKG     68
DREBP1A              ---MCGIKQEM------------SGESSGSPCS-SAS-AERQH-QTVWTAPPKRPAGRTK     42
CRT/DREBP            ----------M------------DT------AA-AGS-PREGH-RTVCSEPPKRPAGRTK     29
OsDREB1F             ------------------EDTSSASSSSVSPPSSPGGGH---HHRLPPKR----RAGRKK     38
CbCBF                ------------------------GSEYESPVSSGGGDYCPTLATSCPKK----PAGRKK     44
BnCBF                ------------------------RSENESPVNTEGGDYI--LAASCPKK----PAGRKK     41
CaCBF1B              ------------------SSSSFSDSSIYSPNRAIFSDEEVILASNNPKK----PAGRKK     52
PaDREB1F             ------------------PQSSLLSDASVTTRGASCSDGDVILASSRPKK----RAGRRV     52
GhDBP                PSYLCQSPQPNTISANSNPMVSFLCPK-------------------------PVPMKHVG     152
GmDREBP_NP           QHV--------------AQTQTHLGPK-------------------------RVPMKHAG     112
ZmDBF1               G----------------------LGPR------------------AQPM-KPAVPVPPAP     33
TINY2                -----------------ERVTQLLVPNSESD----------SVS-DKSKAEQSEKKTKRG     42
ERF034               ------------------------------------KSEEKETNAVCG---SRAVKKQII     42
ZmDBF2               -----------------TRKRQALEAEAEAEA---GGEEEEEEEEGCAGNKAAPAKKRPR     86
ERF038               ------------------RKKEVVGEADEAAGGGAGEEEEEEAEAAAAGKSSAATKKRKR     87
GhDREBP1             -----------------ELGDCCLTS-----------------------SPASGEKRKLH     21
ERF_QDB64575.1       -----------------RRGSGFLKP------------KKKRKSKKGCKGVMSE--GGGD     40
ERF_QCF46602.1       -----------------RRGSKFLKS------------SRKKKKQKEEAGSSAE--KE-S     42
ERF                  -----------------KRGGNLLAS------------LKLNA-YSIAKKSSSKLVKPVS     42
PpDBF1               -----------------KRGGNLLAP------------LKRNV-SSIAKKSSTPK---SG     39
DsDBF1               -----------------KRGGKLLAP------------LK-THSGAIAKKSSAPEKLSIS     42
DREBP5               -----------------KRGGSLLAP------------TLRARSGVIGKKLSSH-----V     38
ERF039-like          -----------------RRRSKR--------------------RVVE---SSDEYPPTSP     38
OsRAP                ----------------------------------------MS-----------SSSPVVA     9
ERF_TINY-like        -----------------SRGTLDIGT------------DTLHCQGLG---RDKQQHAMMK     35
GmDREBP3             ----------------------------------------MAKPSSE---------KP--     9
ERF016               ----------------------------------------MVRPSGG---------RE--     9
ERF016_XP            ----------------------------------------MVRPGRG---------RDNG     11

GmDREBP              GPENLRCNYRGVRQRTW-GKWVAEIREPNR-GSRLWLGTFPTAISAALAYDEAAMAMYG-     115
OsDREBP2A            GPENSNCAYRGVRQRTW-GKWVAEIREPNR-GRRLWLGSFPTALEAAHAYDEAARAMYG-     124
TaDREBP1             GPENSNCAYRGVRQRTW-GKWVAEIREPNR-GNRLWLGSFPTAVEAARAYDDAARAMYG-     125
DREBP1A              FRETRHPVFRGVRRRGNAGRWVCEVRVPGRRGCRLWLGTFDTAEGAARAHDAAMLAINAG     102
CRT/DREBP            FRETRHPLYRGVRRRGRLGQWVCEVRVRGAQGYRLWLGTFTTAEMAARAHDSAVLALLD-     88
OsDREB1F             FRETRHPVYRGVRARAGGSRWVCEVREPQA-QARIWLGTYPTPEMAARAHDVAAIALRGE     97
CbCBF                FRETRHPVYRGVRRRNS-GKWVCEVREPNK-KSRIWLGTFPTADMAARAHDVAAIALRG-     101
BnCBF                FQETRHPIYRGVRLRKS-GKWVCEVREPNK-KSRIWLGTFKTAEIAARAHDVAALALRG-     98
CaCBF1B              FRETRHPVYRGVRKRNS-GKWVCEVREPNK-KSRIWLGTFPTAEMAARAHDVAAIALRG-     109
PaDREB1F             FKETRHPVYRGVRRRNN-DKWVCEMREPNKKKSRIWLGTYPTAEMAARAHDVAALAFRG-     110
GhDBP                APSKPTKLYRGVRQRHW-GKWVAEIRLPRN-RTRLWLGTFDTAEEAALAYDKAAYKLRG-     209
GmDREBP_NP           TAAKPTKLYRGVRQRHW-GKWVAEIRLPKN-RTRLWLGTFDTAEEAALAYDNAAFKLRG-     169
ZmDBF1               APQRPVKLYRGVRQRHW-GKWVAEIRLPRN-RTRLWLGTFDTAEQAALAYDQAAYRLRG-     90
TINY2                RDSGKHPVYRGVRMRNW-GKWVSEIREPRK-KSRIWLGTFPTPEMAARAHDVAALSIKG-     99
ERF034               RDGRKHPTYRGVRMRTW-GKWVSEIREPRK-KSRIWLGTYPTAEMAARAHDVAALAIKG-     99
ZmDBF2               GSEGKHPTFRGVRMRAW-GKWVSEIREPRK-KSRIWLGTFPTAEMAARAHDVAALAIKG-     143
ERF038               SSDGKHPVYRGVRMRAW-GKWVSEIREPRK-KSRIWLGTFPTADMAARAHDVAALAIKG-     144
GhDREBP1             RTQQKEKPFRGIRMRKW-GKWVAEIREPNK-RSRIWLGSYTTPVAAARAYDTAVFYLRG-     78
ERF_QDB64575.1       EGGAPSKTFKGVRKRAW-GKWVSEIREPNK-RSRIWLGSFPTAEMAARAYDAAVVCLRG-     97
ERF_QCF46602.1       EIVPSKTSFKGVRKRAW-GKWVSEIREPNK-RSRIWLGSFPTAEMAARAYDAAVVCLRG-     99
ERF                  LHSGSPKVYKGVRKRTW-GKWVSEIREPNK-RSRIWLGSFPTAEMAAKAYDAAAVCLRG-     99
PpDBF1               KPVGSPKVYKGVRMRTW-GKWVSEIREPNK-RSRIWLGSFPTAEMAAKAYDAAAVCLRG-     96
DsDBF1               QSPACPKLYKGVRMRTW-GKWVSEIREPNK-RSRIWLGSFPTAEMAAKAYDAAVVCLRG-     99
DREBP5               ETQSSSVHYKGVRMRTW-GKWVSEIREPNK-RSRIWLGSFPTAEMAAKAYDAAVVCLRG-     95
ERF039-like          EECDSSPSYKGVRRRSW-GKWVSEIREPRK-RSRIWLGSFATPEMAAKAYDYAVFCLRG-     95
OsRAP                SPPEMEKKYKGVRRRKW-GKWVSEIRLPNS-RDRIWLGSYDSPEKAARAFDAAFVTLRG-     66
ERF_TINY-like        EESTASPRYKGVRKRQW-GKWVSEIREPRK-RSRIWLGSFPTAEMAARAYDAAVVCLRG-     92
GmDREBP3             EEHSDSKYYKGVRKRKW-GKWVSEIRLPNS-RQRIWLGSYDTPEKAARAFDAAMFCLRG-     66
ERF016               ---GNNGRYKGVRMRKW-GKWVAEVRQPNS-RGRIWLGSYKTADEAARAYDAAVVCLRG-     63
ERF016_XP            VNNNSSARYKGVRMRKW-GKWVAEVRQPNS-RRRIWLGSYKTAEEAARAYDAAVFCLRG-     68
                             ::*:* *   .:**.*:*       *:***:: :   ** *.* *   :
```

```
GmDREBP           --FC---ARLNFPNVQVSTFS---EE------PSRNSP------AAAYQS--RNSPSAKE      153
OsDREBP2A         --PT---ARVNFADNSTDANS---GCTSAPSLMMSNGP------A--TIP--SD--EKDE      164
TaDREBP1          --AK---ARVNFSEQSPDANS---GCTLAPPLPMSNGA------TAASHP--SD--GKDE      167
DREBP1A           GGGGGGACCLNFADSAWLLAVPR-----------SYRTLADVRHAVAEAVEDFFRRRLAD      151
CRT/DREBP         -----RAACLNFADSAWRMLPVLAAG------SSRFSSAREIKDAVAIAVLEFQRQRPVV      137
OsDREB1F          --RG---AELNFPDSPSTLPR-----------ARTASP-EDIRLAAAQAAELYRRPPPPL      140
CbCBF             --RS---ACLNFADSAWRLRI-----------PESTGA-KEIQKAAAEAALAFQDEMMMS      144
BnCBF             --RG---ACLNFADSAWRLRI-----------PETTCA-KDIQKAAAEAALAFEAEKSDT      141
CaCBF1B           --RS---ACLNFADSAWRLPV-----------PASSDT-KDIQKAAAEAAEAFRPLKLEG      152
PaDREB1F          --KL---ACINFADSAWRLPV-----------PASMDT-MDIRRAAAEAAEGFRPVEFGG      153
GhDBP             --DF---ARLNFPNLRHHGSH----VGDYKPLPSSVD--AKLQAICESLVQNPKQGSKKK      258
GmDREBP_NP        --EF---ARLNFPHLRHHGAFVFGEFGDYKPLPSSVD--SKLQAICESLAKQEEKPCCSV      222
ZmDBF1            --DA---ARLNFPDNAE----------SRAPLDPAVD--AKLQAICATIAAASSSSKNSK      133
TINY2             --TA---AILNFPELADSFPR-----------PVSLSP-RDIQTAALKAAHMEPTTSFSS      142
ERF034            --QS---AYLNFPDLAHQLPR-----------PATASP-KDIQFAAAKAAAAPKSDGRH-      141
ZmDBF2            --RA---AHLNFPDLAGALPR-----------AASAAP-KDVQAAAALAAAFTSPSS-EP      185
ERF038            --RA---AHLNFPDLAGVLPR-----------AASASP-KDVQAAAALAAAFTTSPSSSP      187
GhDREBP1          --PS---ARLNFPDLIFQED----------------EL-RDISAASIRKKATEVGAKVDA      116
ERF_QDB64575.1    --SN---ATLNFPDSPPSSLL----------LPHCLSP-RDIQAAAAAAAAAAAPPA-DE      140
ERF_QCF46602.1    --SN---ATLNFPNSPPSSL------------PLCGSP-RQIQAAAAAAAAAAAPDP-DE      140
ERF               --QS---VKLNFPDSPPQGV------------QRCTSP-RDVQAAAAAAAAACILPA-SA      140
PpDBF1            --RS---VKLNFPDSPPRCA------------ARCNSP-REVQAAATAAAVACIPSA-TA      137
DsDBF1            --QS---ATLNFPDSPPQCI------------SPSRAP-KDVQAAAAAAAAACASAS---      138
DREBP5            --QS---ATLNFPDSPPQCV------------SPSREP-KDVQAAAAAAAAACCTSS-AT      136
ERF039-like       --PS---AMLNFPDSPPVISP-----------GKKLTSSKDIQAAAAAAAKKKAVPRPAS      139
OsRAP             --HGAAGADLNFPDSPPSCD------------ARSSDP-RQVQAAALSHANRAHVTPQQA      111
ERF_TINY-like     --PS---AALNFPDSPPLSL------------PECHTP-REIQVVAAAAATAAESCTPLN      134
GmDREBP3          --RN---AKFNFPDNPPDIAG-----------GTSMTP-SQIQIAAAQFANAGPHEG---      106
ERF016            --SS---ATLNFPDNPPEIPL-----------ADEMTP-VQIQEFAFRHARRAPAQS---      103
ERF016_XP         --SS---GKLNFPDNPPDIAD-----------ASELTP-EQIQEAAFRHARKDITEE---      108
                           .** .

GmDREBP           SGSALVILERSECMMLWNNSGGDAAEDD---------G-----MED------LSLSLSVK      193
OsDREBP2A         LESPPFIVANGPAVLYQ-PDKKDVLERV---------V-----PEVQDVKTEGSNGLKRV      209
TaDREBP1          SESPPSLISNAPTAALHRSDAKDESESA---------G-----TVARKVKKEVSNDLRST      213
DREBP1A           DALSATS------------SSSTTP---------STPRTDDDEESAATDGDESSSPAS--      188
CRT/DREBP         ST----------------------------------SEMHDGEKDAQGSPTPSELSTS--      161
OsDREB1F          ALP--------------------------------------------EDPQEGTS-----      151
CbCBF             --------------------DTTTT--D--HGFDMEETFVE--AIVTAEQ----------      168
BnCBF             TTNDHGMNMA-----S-QVEVNDTT--D--HDLDMEETIVE--AVFREEQREGFYMAE--      187
CaCBF1B           ISKESSS---------------------------S-------------------------      160
PaDREB1F          VCSGSSD---------------------------EKERMVV--QV-EEKNKKGSVN----      179
GhDBP             SS-KVTADTK----SR-NNKKSDMAE-----PKPEEN----------TAKVENSSSLS--      295
GmDREBP_NP        EDVKPVIHAA----EL-AEVESDVAKSNAEYVYPEFE----------DFKVEHENPMF--      265
ZmDBF1            AKSKAMPINA----S--------VLEAAA--ASPSN-----------SSSDEGSGSGF--      166
TINY2             STSSS--------SSL-SSTSS-LE--SLVL------V---------MDLSR--T-----      168
ERF034            -----------------------------------------------SPADR--AP----      148
ZmDBF2            GAG------------A-HEEP-------AAK------D---------GAAPE--------      202
ERF038            SSS------------L-SADD--VA--PCVV------H---------ADADE--QPAAAA      213
GhDREBP1          LQTS-LHHAS----AS-SSESS-NP--TRVFRKPDLNKYPD-------SSDED-------      153
ERF_QDB64575.1    GSTPSSSTVP----S-----AS-PP--PRPN--------------------------S--      160
ERF_QCF46602.1    ASNPASDPAS----SQ-PDEGS-NP--TRPFSIPELGIVKR--EAFDSQPNEGSNPAKPF      190
ERF               LPMVN-HS-V-----T-PSFES-TP--LN--SLHSSSGMSS------DDEAEDIDRSHHV      181
PpDBF1            LTVAN-FQSP-----A-QTLES-SP--LH--SSPSSSDMSS------GDEDSGEDSVQSF      179
DsDBF1            -PLA--EPTN-----T-PTFES-TT--V--ESLHSPLHC----------EA---------      165
DREBP5            PSIG--EPAN-----I-PTFES-SS--SAVESFSASSSQ----------EA---------      166
ERF039-like       VTSKPV---------------------------PLPSSS--------KQAQEEEAPVTPL      164
OsRAP             AAAL-------------------MS--P-----PSLSPP--------PGFATGSEVVAPA      137
ERF_TINY-like     FQVKEEAQSLGFQLSI-RNETN-VS--PRSHLRPVESEN--------SSTSEGRS-----      177
GmDREBP3          -----------------------HS--GRPEHPPMESPS--------PSVSEGTI-----      128
ERF016            -----------------------VE--DLGSNEA---AV---------------------      114
ERF016_XP         -----------------------AE--VKEEMAA---GG---------------------      119
```

```
GmDREBP           HEE-----------------------GE---DESGTS-----SSYLSLS-----------    211
OsDREBP2A         CQERKNMEVC-------------ESEGIVLHKEVNIS-----YDYFNVHEVV-----EMI    246
TaDREBP1          HEEHKTLEVS-------------QPKGKALHKAANVS-----YDYFNVEEVL-----DMI    250
DREBP1A           ----------------------------------DLAFELDVLSDMGWDLYYASLAQGML    214
CRT/DREBP         ----------------------------------SDLLDEHWFGGMDAGSYYASLAQGML    187
OsDREB1F          -----------GGG----ATATSGRPAAVFVD---------EDAIFDMPGLIDDMARGMM    187
CbCBF             -------------------------SASLYID---------EEDMFGMPSLMASMAEGML    194
BnCBF             --ET-----T-VVG----VVPEEQMSKGFYMD---------EEWMFGMPTLLADMAAGML    226
CaCBF1B           -----------------------TPESMFFMD---------EEALFCMPGLLTNMAEGLM    188
PaDREB1F          -------------------LERSRSLSLSYWD---------EEEVFHMPRLLHDMAEGLL    211
GhDBP             ------------------------------TVQSESE--------------GSAV-----    306
GmDREBP_NP        ---------------------------------------------------SGES-----    269
ZmDBF1            ------------------------------GSDDE-M--------------SSSS-----    176
TINY2             -E---SEELGEIVELPSLGAS-YDVDSANLGNE---------FVFYD-------------    201
ERF034            ----------------------SSPTADNDDDD---------AALFDLPDLL--------    169
ZmDBF2            -EAAADAQAPVPVALPPPAASRPGTPSSGVEDE---------RQLFDLPDLL--------    244
ERF038            KNDDDDGSTTAPV---------AAAAAAAAADE---------QQLFDLPDLL--------    247
GhDREBP1          ------------------------------------------------------------    153
ERF_QDB64575.1    --------------------PVKKEVQ-EDGEDDH-----NLQGLFDMPMSPEDL-----    189
ERF_QCF46602.1    SIELG----------HE-EGTVKREIM-E--EEDNHLQGLLMDGILNLPLSPDF------    230
ERF               TKF---CS-------HVDVVPVE-------------------------------------    194
PpDBF1            VAEGSQIS-------PSEVVPVLEWVKVEFGDK-----ETVAAGDVDFDCDYSFI-----    222
DsDBF1            SSALAMAE-------PQSSFEVEEWIQAEFGDL-----EPLIDNAFRFPELPPC------    207
DREBP5            SSAVT-LT-------SSSTVPLEEWINSEFGEL-----EPLLDNAFRFPELPPL------    207
ERF039-like       LVSE-----------SSPSSLRRTWSL-------QLDEDFAAMA-MLLPPLPPLP-----    200
OsRAP             VRADGSIDWRPVMAHPPPLYSPPGWGG-------GHAYDFLQPP-------PPSP-----    178
ERF_TINY-like     -RKI---DLS--------------SKD-------EHDEELDYTG-------ESCP-----    200
GmDREBP3          -QTDS--DVP--------------TLN-------GSVTD-LFTP-------VGSS-----    151
ERF016            ----------------------------------------VSAS-------GSSS-----    122
ERF016_XP         ----------------------------------------NFNG-------ECYF-----    127

GmDREBP           ------------------------------------------------------------    211
OsDREBP2A         IVELSADQKTE--------------VHEEYQ---EGD-----------------------    266
TaDREBP1          IVELSADVKME--------------AHEEYQ---DGD-----------------------    270
DREBP1A           MEPPSAALG--DDGD-----------------------AILADVPLWSY-----------    238
CRT/DREBP         MEPPSARTWSEDGGE-----------------------YSAVYTPLWN------------    212
OsDREB1F          LTPPAIGRSLDDWAAID-----------------DDDDHYHMDYKLWMD-----------    219
CbCBF             LPLPSVQ--WNHNYDID-----------------GDDD-----VSLWSY-----------    219
BnCBF             LPLPSVQ--WGHNDDFE-----------------GDAD-----MNLWNY-----------    251
CaCBF1B           LPPPQCAEIGDHV--ET-----------------ADAD-----TPLWSYSI---------    215
PaDREB1F          LSPSQCLGGYMNLDDMG-----------------TDAD-----VKLWSFSI---------    240
GhDBP             -SSPLSDLT---FSDFDE---QPWPEVVSSSE-TFMLSKYP-SEIDWDSILKA-------    350
GmDREBP_NP        -SSPESSVTFLDFSDFSDS-NNQWDE----ME-NFGLEKFPSVEIDWEAI----------    312
ZmDBF1            -PTPVVAPPVADMGQLDFS-EVPWDE----DE-SFVLRKYPSYEIDWDALLSN-------    222
TINY2             -------------SVDYCLYPPPWGQSSEDNYGHGISPNF-GHGLSWDL-----------    236
ERF034            ----------LDLKWSSW------LSSSSSE--YFAEIRLDHEPFLWEPFQLGENIHVFD    211
ZmDBF2            ----------LDIRDGFGRFPPMWAPLTDVEDVVNAELRL-EEPLLWE------------    281
ERF038            ----------FDIQDGPFGFPAMWAPLADVDE-VNAELRL-EEPLLWDLGVTDA------    289
GhDREBP1          ------------------------------------------------------------    153
ERF_QDB64575.1    -ALPAAAM----------------------EF-APSTSEFWELDNLWSFSR---------    216
ERF_QCF46602.1    --IPVAAI----------------------EP-AAAASEFWELDNLWGFAR---------    256
ERF               ------------------------------------------------------------    194
PpDBF1            -SEFAADS------LLACSYNSKG--RTEYLP-IETHDAALYDSTLWFF-----------    261
DsDBF1            --------------VFDSQFQI-F--QPSAGP-LEADNRALYDS-LWCFS----------    238
DREBP5            --------------DQICQ-------LPSGAF-LDTESRMLYDS-LWCFS----------    234
ERF039-like       -PIQMTD------------------------E-EDSDHGVLEHLDLW-------------    221
OsRAP             -PLPSCDDDMVDVVD-------------------------ESSASLWSFDTRD-------    205
ERF_TINY-like     -PLLTADDYYVSLDDVELAWDNMKVDDL----------NFLDLPPLEDFFEDGPRKLTKT    249
GmDREBP3          -GYASDYGIFPGFDDFSGDFYVPEMPNVNYGE-ENGEGFIVDESFLWNF-----------    198
ERF016            -GYGVDD------V--------------------GVDGAFYQSPGLWTF-----------    144
ERF016_XP         -GGGSVG------D--------------------GSGGTYYHSSGFWTF-----------    149
```

```
GmDREBP           ---------------------------------   211
OsDREBP2A         --DGFSLFSY-----------------------   274
TaDREBP1          --DGFSLFSY-----------------------   278
DREBP1A           ---------------------------------   238
CRT/DREBP         ---------------------------------   212
OsDREB1F          ---------------------------------   219
CbCBF             ---------------------------------   219
BnCBF             ---------------------------------   251
CaCBF1B           ---------------------------------   215
PaDREB1F          ---------------------------------   240
GhDBP             ---------------------------------   350
GmDREBP_NP        ---------------------------------   312
ZmDBF1            ---------------------------------   222
TINY2             ---------------------------------   236
ERF034            L---HSLV-------------------------   216
ZmDBF2            ---------------------------------   281
ERF038            ---------------------------------   289
GhDREBP1          ---------------------------------   153
ERF_QDB64575.1    ---------------------------------   216
ERF_QCF46602.1    ---------------------------------   256
ERF               ---------------------------------   194
PpDBF1            ---------------------------------   261
DsDBF1            ---------------------------------   238
DREBP5            ---------------------------------   234
ERF039-like       ---------------------------------   221
OsRAP             -----SYFRY-----------------------   210
ERF_TINY-like     NSGRQSLVQYCRQFSSNYERVDSMHLVCDVFTM   282
GmDREBP3          ---------------------------------   198
ERF016            ---------------------------------   144
ERF016_XP         ---------------------------------   149
```

Figure S2. Sequence alignments of DsDBF1 and other known ERF/DREB proteins from mosses and vascular plants such as *Syntrichia caninervis* (DREBP5_AMT92109.1), *Physcomitrium patens* (ERF RAP2-1-like_XP 024372564.1; PpDBF1 ABA43687.2; ERF_TINY-like_XP 024390306.1), *Gossypium hirsutum* (GhDREBP1_AAO43165.1; GhDBP_RAP2-4-like_NP 001314591.1), *Glycine max* (GmDREBP3_ABB36646.1; GmDREBP_NP 001345276.1; GmDREBP_NP 001345278.1), *Oryza sativa* (OsRAP_XP 468111.1; OsDREBP2A_XP 025878770.1; DREBP1A_XP 015610912.1; OsDREB1F_NP 001359120.1; ERF038_XP 015614793.1), *Triticum aestivum* (TaDREBP1_AAL01124.1; CRT/DREBP_XP 044398325.1), *Zea mays* (ZmDBF1_AAM80486.1; ZmDBF2_AAM80485.1), *Capsicum annuum* (CaCBF1B_AAQ88400.1), *Capsella bursa-pastoris* (CbCBF_AAR26658.1), *Brassica napus* (BnCBF_AAL38243.1), *Prunus avium* (PaDREB1F_XP 021803652.1), *Arabidopsis thaliana* (TINY2_NP 196720.1), *Bryum argenteum* (ERF_QDB64575.1), *Pohlia nutans* (ERF_QCF46602.1), *Selaginella moellendorffii* (ERF039-like_XP 024530345.1), *Citrus sinensis* (ERF016_XP 006474696.1), *Apostasia shenzhenica* (ERF034_PKA61103.1) and *Theobroma cacao* (ERF016_XP 007012585.2). Multiple alignment was performed using Clustal Omega. Amino acid sequences are highlighted with different colors. Sequences marked by (*) show conserved amino acid residues.
