# Supplementary material for: DsDBF1, a Type A-5 DREB Gene, Identified and Characterized in the Moss Dicranum scoparium
Source: Life (Basel). 2022 Dec 28;13(1):90. doi: 10.3390/life13010090 (PMC9862540; doi:10.3390/life13010090)
Supplement: Supplementary file 1 [file life-13-00090-s001.zip › Table S1.pdf]

Table S1. Primers of RT-qPCR.

Gene name	Sequence 5'–3'	Product size, bp	Tm, °C
DsDBF1	F: TGGGTGTCTGAGATTCGAGAG<br>R: GTAGGCTTTGGCTGCCATT	87	60
18S	F: TGGAGGGCAAGTCTGGTG<br>R: TTTAACTGCAACAACTTAAATATACGC	75	60
GAPDH2	F: TCAGTGGTGGACTTGACTGTG<br>R: CTGCCTTGACGTCCTCGTA	61	59
a-TUB1	F: TCTATGATGGCGAAGTGCGACCCC<br>R: GGGGGCTGGTAGTTGATTCCGCA	182	64
a-TUB2	F: TTGTGGACTTGTGCTTGGACCG<br>R: AGCCTCTCCAGCAACAGCGAAC	130	59
